# Sequencing of BRAF inhibitors and ipilimumab in patients with metastatic melanoma: a possible algorithm for clinical use

**DOI:** 10.1186/1479-5876-10-107

**Published:** 2012-05-28

**Authors:** Paolo A Ascierto, Ester Simeone, Diana Giannarelli, Antonio M Grimaldi, Anna Romano, Nicola Mozzillo

**Affiliations:** 1 Melanoma, Immunotherapy and Innovative Therapy Unit, Istituto Nazionale Tumori Fondazione “G. Pascale”, Naples, Italy; 2 Biostatistical Unit, Scientific Division, Regina Elena Cancer Institute of Rome, Rome, Italy; 3 Unit of Medical Oncology and Innovative Therapy, Istituto Nazionale per lo Studio e la Cura dei Tumori “Fondazione G. Pascale”, Via Mariano Semmola 80131, Napoli, Italia

**Keywords:** Dabrafenib, Disease progression, Ipilimumab, Treatment sequencing, Vemurafenib

## Abstract

**Background:**

Ipilimumab and vemurafenib have both been shown to improve survival in phase III trials of patients with metastatic melanoma. Although vemurafenib is associated with a rapid onset of activity, responses are often of limited duration. Conversely, responses to ipilimumab take time to develop, but can be durable. Currently, limited data exist on the sequencing of these agents in patients with the BRAF^V600^ mutation. The aim of this analysis was to identify factors that could potentially be used to optimise the order in which ipilimumab and BRAF inhibitors are administered in this patient population.

**Methods:**

This was a retrospective, single-institution, analysis of patients treated with vemurafenib 960 mg or dabrafenib 150 mg twice-daily and ipilimumab 3 mg/kg every 3 weeks for 4 doses as part of a clinical trial or expanded access program. Eligible patients tested positive for the BRAF^V600^ mutation and had sequentially received treatment with vemurafenib or dabrafenib followed by ipilimumab, or vice versa.

**Results:**

In total, 34 BRAF-mutation positive patients were eligible, comprising six patients who received ipilimumab followed by a BRAF inhibitor, and 28 patients treated with a BRAF inhibitor who subsequently received ipilimumab. Of these 28 patients, 12 (43 %) had rapid disease progression resulting in death and were unable to complete ipilimumab treatment as per protocol. These patients were classified as having rapid disease progression. Median overall survival for rapid progressors was 5.7 months (95 % CI: 5.0–6.3), compared with 18.6 months (95 % CI: 3.2–41.3; p < 0.0001) for those patients who were able to complete ipilimumab treatment. Baseline factors associated with rapid progression were elevated lactate dehydrogenase, a performance status of 1 and the presence of brain metastases. Patients were more likely to have rapid disease progression if they had at least two of these risk factors at baseline.

**Conclusions:**

Our analysis suggests it may be possible to identify those patients at high risk of rapid disease progression upon relapse with a BRAF inhibitor who might not have time to subsequently complete ipilimumab treatment. We hypothesise that these BRAF-mutation positive patients may benefit from being treated with ipilimumab first.

## Introduction

Until recently, patients with metastatic melanoma had limited treatment options and a very poor prognosis. In a meta-analysis of 42 phase II trials with 2,100 patients, median survival was approximately 6 months, and only a quarter of patients were alive after one year [1].

Despite many efforts over the past 30 years to improve outcomes, no treatment was shown to improve survival in metastatic melanoma [2]. However, due to significant advances in our understanding of cancer immunology and the molecular pathways involved in melanoma pathogenesis, the treatment landscape for metastatic melanoma has, in recent times, undergone dramatic changes.

The recent approvals of vemurafenib and ipilimumab means that physicians are now equipped with tools that will allow some patients with metastatic melanoma to live longer [3-5]. However, while both drugs have well-documented benefits, they also have significant limitations. Although treatment with BRAF inhibitors, such as vemurafenib and dabrafenib, can result in the rapid onset of tumour response in many patients, intrinsic and/or acquired resistance means these are often temporary, with a median time to progression of less than 7 months [5]. Furthermore, results from clinical trials of vemurafenib suggest that progression can be rapid in some patients. In the BRIM2 trial, among 39 patients that died as a result of disease progression, 16 (41 %) died within 28 days of their last dose of vemurafenib [6]. In BRIM3, of 42 vemurafenib-treated patients who died during the course of the study, 22 (52 %) died within 28 days of their last dose, with almost all deaths attributed to disease progression [7]. By contrast, although ipilimumab has a slow onset of effect and a low rate of objective responses, long-term follow-up from clinical trials has demonstrated that responses can be durable [8,9]. The two classes of agent therefore have very different, but potentially complimentary profiles, supporting a combination or sequencing approach to treatment.

Evidence suggests that BRAF inhibition and immunotherapy may act synergistically. In preclinical studies, T-cell viability and function was preserved when peripheral blood mononuclear cells and BRAF^V600E^ mutant melanoma cells were exposed to clinically relevant concentrations of vemurafenib in vitro [10]. In addition, an analogue of vemurafenib was shown to increase both antigen presentation by melanoma cells and their recognition by melanoma-specific T cells [11]. Together, these studies support the rationale that inhibition of BRAF^V600^ could render melanoma cells more susceptible to attack by immunotherapeutic strategies. However, further investigations are required to determine how the agents can be best used together to optimise outcomes in those patients with a BRAF^V600^ mutation.

One strategy may be to use the two drugs sequentially; for example, to start with a BRAF inhibitor to reduce the tumour load, then use ipilimumab to maintain the response; or start with ipilimumab and provide vemurafenib afterwards to reduce the tumour burden.

Preclinical and clinical studies investigating the combination of immunotherapy and chemotherapy have highlighted that the sequence in which the agents are administered can affect outcome [12,13]. The aims of this retrospective study were to determine if the sequence in which the BRAF inhibitors vemurafenib and dabrafenib were administered with ipilimumab had an effect on clinical outcome and to identify predictive factors that could potentially be used to guide decisions regarding treatment.

## Methods

### Patients

This was a single-institution, retrospective analysis of patients treated within clinical trials or as part of an expanded access program (EAP) at the National Cancer Institute, Naples, Italy.

Patients were eligible for analysis if they tested positive for the BRAF^V600^ mutation and had sequentially received vemurafenib or dabrafenib and ipilimumab, or vice versa.

Patients could have received vemurafenib 960 mg twice daily within the phase III BRIM3 study (NCT01006980) [3] if they had previously untreated, unresectable, stage IIIC or stage IV (metastatic) melanoma; or within the phase III vemurafenib EAP (NCT01307397) [14] if they had previously untreated or pretreated metastatic melanoma.

Treatment naïve or previously treated patients with metastatic melanoma could have received dabrafenib 150 mg twice daily within the phase II BREAK-2 trial (NCT01153763) [15] or within the phase II BREAK-MB trial (NCT01266967) if their melanoma had metastasised to the brain [16].

Ipilimumab 3 mg/kg was administered intravenously every 3 weeks for 4 doses as part of the ipilimumab EAP (NCT00495066) for patients aged ≥ 16 years with unresectable stage III/stage IV melanoma who had either failed systemic therapy or were intolerant to ≥ 1 systemic treatment and for whom no other therapeutic option was available [17]. For patients treated with ipilimumab, tumour assessments were performed according to immune-related response criteria [18].

The protocols for the aforementioned studies were approved by the institutional review board of the National Cancer Institute, Naples, Italy and the studies were all conducted in accordance with the ethical principles of the Declaration of Helsinki and within the Good Clinical Practice guidelines, as defined by the International Conference on Harmonization. All patients provided written informed consent before enrollment, where applicable.

### Statistical analysis

Based on observations from the BRIM2 and BRIM3 clinical trials that 40–50 % of vemurafenib-treated patients who died as a result of disease progression died within 28 days of the last vemurafenib dose [6,7], the primary aim of this retrospective study was to identify baseline factors that could be used to predict which BRAF inhibitor-treated patients would experience rapid disease progression upon relapse. Rapid progression was defined as not surviving long enough to subsequently complete all four induction doses of ipilimumab.

Patient and disease characteristics were summarised using relative frequencies (percentages) for categorical variables and median for continuous variables. Comparisons between the groups were performed using a two-sided chi-square test. A logistic regression model was used to determine if the following factors could be used to predict which patients would complete ipilimumab induction therapy: gender, age, Eastern Cooperative Oncology Group performance status (ECOG PS), lactate dehydrogenase (LDH) level, presence/absence of brain metastases, previous lines of therapy and the BRAF inhibitor used. Progression-free survival and overall survival were estimated using the Kaplan-Meier product-limit method, with differences between curves evaluated using the log-rank test.

## Results

### Patients and treatment

In total, 34 BRAF-mutation positive patients were treated sequentially with a BRAF inhibitor and ipilimumab, comprising six patients who received a BRAF inhibitor upon disease progression with ipilimumab and 28 patients who received ipilimumab upon disease progression with a BRAF inhibitor. Among the 28 patients treated with a BRAF inhibitor first, 12 (43 %) received vemurafenib and 16 (57 %) received dabrafenib. Among the six patients who received ipilimumab first, two (33 %) went on to receive dabrafenib and four (67 %) received vemurafenib. Baseline characteristics of the 34 patients are summarised in Table 1. Characteristics were mostly comparable between the two groups, although all patients in the ipilimumab-first group had an ECOG PS of 0 compared with 50 % of patients in the BRAF inhibitor-first group. Approximately two-thirds of patients included in the analysis were male, half had elevated LDH and most (31 out of 34; 91 %) were metastatic stage M1c. All six patients treated with ipilimumab first had received prior treatment; therefore, ipilimumab represented the second line of therapy in each case. Of patients treated with a BRAF inhibitor, half had received prior therapy. Overall, prior therapy comprised chemotherapy in 70 % of patients (n = 14) and immunotherapy with MAGE-A3 or targeted therapy with a MEK inhibitor in 15 % of patients, respectively (n = 3 for each).

**Table 1:** Baseline characteristics of patients treated sequentially with BRAF inhibitors and ipilimumab

**Characteristic**	**BRAF inhibitor followed by ipilimumab (n = 28)**	**Ipilimumab followed by a BRAF inhibitor (n = 6)**
BRAF inhibitor, n (%)		
Vemurafenib	12 (43)	4 (67)
Dabrafenib	16 (57)	2 (33)
Median age, years	50	48
Male/female, n (%) / n (%)	18 (64) / 10 (36)	4 (67) / 2 (33)
ECOG PS		
0	15 (54)	6 (100)
1	13 (46)	0 (0)
LDH level, n (%)		
<1.10 ULN	14 (50)	3 (50)
≥1.10 ULN	14 (50)	3 (50)
Disease stage, n (%)		
Unresectable IIIc	1 (4)	0 (0)
M1b	2 (7)	0 (0)
M1c	25 (89)	6 (100)
Brain metastasis, n (%)	7 (25)	3 (50)
Previous therapy, n (%)	14 (50)	6 (100)
Mage-A3	2 (7)	1(17)
Dacarbazine	5 (18)	1 (17)
Temozolomide plus cisplatin	2 (7)	2 (33)
Cisplatin, vinblastine and dacarbazine	3 (11)	0 (0)
Fotemustine	0 (0)	1 (17)
MEK inhibitor	2 (7)	1 (17)

ECOG PS, Eastern Cooperative Oncology Group performance status; LDH, lactate dehydrogenase; ULN, upper limit of normal.

### Treatment with ipilimumab followed by a BRAF inhibitor

All six patients were alive at the time of analyses with a median follow-up of 11.2 months. Tumour responses achieved with ipilimumab and subsequently with vemurafenib or dabrafenib are provided in Table 2. Of the six patients, three patients (50 %) achieved immune-related disease control (complete response [CR], partial response [PR] or stable disease [SD]) with their initial ipilimumab treatment, and all six attained disease control (a PR in five patients and SD in one) upon subsequent treatment with a BRAF inhibitor.

**Table 2:** Summary of tumour response and median time to progression

	**BRAF inhibitor followed by ipilimumab (n = 28)**			**Ipilimumab followed by a BRAF inhibitor (n = 6)**		
	**Vemurafenib**	**Dabrafenib**	**Ipilimumab**	**Ipilimumab**	**Vemurafenib**	**Dabrafenib**
Patients, n	12	16	28	6	4	2
Objective tumour response*, n (%)	4 (33)	10 (63)	7 (25)	1 (17)	3 (75)	2 (100)
CR	0 (0)	1 (6)	0 (0)	0 (0)	0 (0)	0 (0)
PR	4 (33)	9 (56)	7 (25)	1 (17)	3 (75)	2 (100)
SD	4 (33)	1(6)	7 (25)	2 (33)	1 (25)	0 (0)
PD	4 (33)	5 (31)	7 (25)	3 (50)	0 (0)	0 (0)
Median time to progression, months (95 % CI)	3.6 (3.3–3.8)	4.0 (2.1–5.9)		3.4 (2.8–4.1)		

*Determined at Week 12 among ipilimumab-treated patients according to immune-related response criteria [18].

CI, confidence interval; CR, complete response; PD, progressive disease; PR, partial response; SD, stable disease.

The median time to disease progression with ipilimumab treatment was 3.4 months (Table 2), which corresponded exactly with the median time from progression to initiating treatment with a BRAF inhibitor, suggesting none of the patients had rapidly progressing disease.

### Treatment with a BRAF inhibitor followed by ipilimumab

Tumour responses achieved with vemurafenib or dabrafenib and subsequently with ipilimumab are provided in Table 2. Eighteen patients achieved disease control with BRAF inhibition (64 %), comprising one CR, 13 PRs and five patients with SD. Upon subsequent treatment with ipilimumab, the immune-related disease control rate was 50 %, with seven patients each achieving a PR or SD.

Median time to disease progression was 3.6 months for vemurafenib and 4 months for dabrafenib. However, the median time from disease progression with a BRAF inhibitor to starting treatment with ipilimumab was just 28 days.

Among the 28 patients, 12 had rapid disease progression resulting in death and were unable to complete all four induction doses of ipilimumab 3 mg/kg as per protocol. For these patients, overall survival was 5.7 months (95 % CI: 5.0–6.3). The remaining 16 patients had slower disease progression and were able to complete induction therapy with ipilimumab. Median overall survival for these patients was significantly longer at 18.6 months (95 % CI: 3.2–41.3; p < 0.0001). Median overall survival for all 28 patients was 14.3 months (95 % CI: 4.8–23.8).

**Figure 1:**
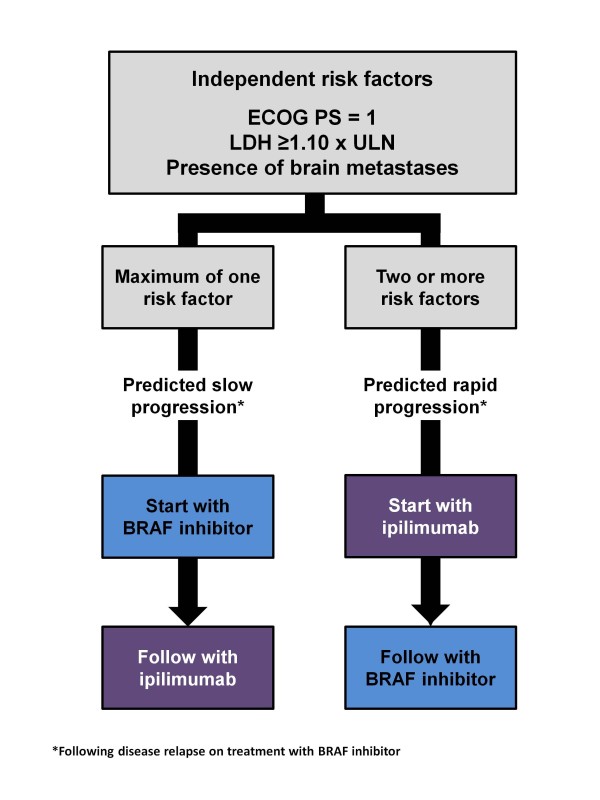
**Suggested algorithm for the sequential use of ipilimumab and BRAF inhibitors in patients with metastatic, BRAF**^**V600**^**mutation-positive melanoma.** Abbreviations: ECOG PS, Eastern Cooperative Oncology Group Performance Status; LDH, lactate dehydrogenase; ULN, upper limit of normal.

The two groups of patients, subsequently classified as rapid progressors or slow progressors, respectively, were analysed according to baseline factors (Table 3). Univariate analysis highlighted that being < 50 years of age, an ECOG PS of 1, LDH level ≥ 1.10 times the upper limit of normal (ULN) and the presence of brain metastases were all significantly associated with a poorer outcome; i.e. with not completing the entire ipilimumab induction regimen. In a multivariate analysis, LDH level and the presence of brain metastases remained significant. Furthermore, including ECOG PS in the model increased the rate of correct classifications to 93 %, suggesting that these three factors could be independent risk factors for rapid progression (Figure 1).

**Table 3:** Univariate analysis showing correlation between baseline factors and completion of ipilimumab induction therapy (3 mg/kg every 3 weeks for a total of four doses)

**Characteristic**	**Slow progressors n (%)**	**Rapid progressors n (%)**	***P* value**
Gender			
Male	10 (56)	8 (44)	0.82
Female	6 (60)	4 (40)	
Age			
<50 years	5 (36)	9 (64)	0.02
≥50 years	11(79)	3 (21)	
ECOG PS			
0	12 (80)	3 (20)	0.009
1	4 (31)	9 (69)	
Previous lines of therapy			
0	9 (64)	5 (36)	0.44
1	7 (50)	7 (50)	
Brain metastasis			
Yes	0 (0)	7 (100)	<0.0001
No	16 (76)	5 (24)	
LDH			
<1.10 ULN	13 (93)	1 (7)	<0.001
≥1.10 ULN	3 (21)	11 (79)	
BRAF inhibitor			
Vemurafenib	7 (58)	5 (42)	0.91
Dabrafenib	9 (56)	7 (44)	

ECOG PS, Eastern Cooperative Oncology Group performance status; LDH, lactate dehydrogenase; ULN, upper limit of normal.

Additional analysis demonstrated a correlation between the number of risk factors and completion of ipilimumab induction. Among patients treated with a BRAF inhibitor prior to receiving ipilimumab, a maximum of one risk factor was associated with slow progression, while the presence of two or more risk factors was associated with rapid progression (Table 4).

**Table 4:** Correlation between number of baseline risk factors and completion of ipilimumab induction therapy (3 mg/kg every 3 weeks for a total of four doses)

	**Number of risk factors**			
	**0**	**1**	**2**	**3**
Received BRAF inhibitor first and ipilimumab upon disease progression (n = 28)				
Slow progressors (n = 16)	11	3	2	0
Response to ipilimumab	PR (n = 3); SD (n = 6); PD (n = 2)	PR (n = 3)	PR (n = 1); PD (n = 1)	-
Rapid progressors (n = 12)	0	1	7	4
Response to ipilimumab	-	SD (n = 1)	NE (n = 4); PD (n = 3)	NE (n = 3); PD (n = 1)
Received ipilimumab first and a BRAF inhibitor upon disease progression (n = 6)				
Completed induction regimen (n = 6)	2	2	2	0
Response to ipilimumab	PR (n = 1); PD (n = 1)	PD (n = 2)	SD (n = 2)	-

NE, not evaluable; PD, progressive disease; PR, partial response; SD, stable disease.

## Discussion

For patients with BRAF-mutation positive metastatic melanoma, vemurafenib and ipilimumab both represent important approved treatment options. A phase III trial of dabrafenib compared with dacarbazine has also recently completed (NCT01227889) [19], with results imminent. Following results from a phase I/II trial that showed the combination of dabrafenib and trametinib, a MEK inhibitor, had antitumour activity and a decreased incidence of skin-related adverse events than dabrafenib alone [20,21], randomised phase III trials comparing this combination with dabrafenib alone (NCT01584648) or vemurafenib alone (NCT01597908) are planned.

Treatment guidelines for metastatic melanoma stress the importance of screening patients for mutations and recommend that vemurafenib is preferentially used in patients with BRAF^V600^ mutation-positive melanoma who have symptomatic disease [22]. Vemurafenib is not indicated for patients with wild-type BRAF [23]. By contrast, ipilimumab can be used to treat patients with metastatic melanoma, regardless of their BRAF status. In a retrospective analysis of tumour biopsies from patients treated with ipilimumab in a phase II clinical trial, rates of objective responses and stable disease in patients with BRAF^V600E^ mutation-positive tumours were comparable with those in patients with the wild-type gene [24].

Historically, oncologists were used to responses to conventional anticancer therapies like chemotherapy occurring within days or weeks of starting treatment. Importantly, ipilimumab is associated with unique patterns of response, related to its mechanism of action, which can influence treatment choice. Because it can take weeks to months to build a complete immune response against a tumour, responses with ipilimumab may not be detectable until Week 12 of treatment. Furthermore, during this period the cancer may progress or appear to progress [18,25]. Because the timing of responses is different, ipilimumab treatment may not be appropriate for those patients who have rapidly progressing disease. If a physician considers that their patient will not have time to complete the 12-week induction course and wait for a response, then other treatment options should be considered [26]. Although clinical success is noted for vemurafenib, treatment with this agent as the first stage of a sequential strategy may also present difficulties. Even if a patient initially responds to the BRAF inhibitor, they may subsequently relapse and progress. If disease progression is rapid, the patient may not have the time available to them to respond to subsequent immunotherapy [26].

Previous studies have highlighted an older age, poor performance status and male gender to be associated with poor prognosis in patients with melanoma [27]. More recently, elevated LDH levels have also emerged as a significant negative prognostic indicator [28,29]. Historically, those patients with advanced disease and brain metastases have also had a particularly poor prognosis, with a life expectancy of only 3–5 months [30,31].

Nevertheless, there is now some renewed hope for these patients. Subgroup analyses of clinical trials have suggested that the effect of ipilimumab and vemurafenib on overall survival is independent of age, gender, baseline LDH and metastatic stage of disease [3,4,26]. Furthermore, preliminary studies suggest ipilimumab and BRAF inhibitors may also have activity in patients with melanoma brain metastases [32-34]. These findings indicate that patients with baseline characteristics associated with high-risk, symptomatic disease can potentially benefit from treatment with both BRAF inhibitors and ipilimumab [4,26]. The question our study aimed to address was, based on baseline factors; can we determine which patients are more likely to have rapid disease progression after developing resistance to BRAF inhibitors and would therefore be less likely to be able to receive subsequent treatment with ipilimumab? That is, can we determine the optimal sequence in which these agents should be used?

In this retrospective analysis, among 28 patients treated with a BRAF inhibitor first, almost half were unable to complete treatment with ipilimumab due to rapid disease progression. The most significant risk factors for rapid progression were elevated LDH, a PS of 1 and the presence of brain metastases. Although this was a retrospective study of a small number of patients, the data suggest that the presence of two or more of these risk factors may predict for rapid disease progression. Our hypothesis is that patients with two or more risk factors could potentially benefit from receiving ipilimumab as the first part of their sequential treatment regimen (Figure 1). It is important to note, however, that among the six patients who received ipilimumab first and were subsequently treated with a BRAF inhibitor upon disease progression, there was no correlation between the number of risk factors at baseline and rate of progression (Table 4).

Interestingly, in a post-hoc analysis of patients from BRIM3 treated with vemurafenib, there was a 50 % reduction in the risk of death for patients with LDH greater than the ULN, and a 48 % reduction in the risk of death for patients with an ECOG PS of 1. By comparison, the risk of death was reduced by 35 % among patients with normal LDH and 36 % for patients with an ECOG PS of 0 [23]. These results suggest that the benefit of vemurafenib is greater in patients with negative prognostic factors, which is perhaps unsurprising as patients with the poorest prognosis would potentially have the most to gain from treatment.

It is possible that patients who are at risk of rapid disease progression according to our proposed algorithm would benefit from concomitant treatment with ipilimumab and a BRAF inhibitor. The safety and efficacy of ipilimumab and vemurafenib combination therapy is currently being assessed in a prospective, multicenter phase I/II trial (NCT01400451) of patients with BRAF ^V600^ mutation-positive metastatic melanoma to determine whether additional benefits are possible with combination therapy compared with the use of either agent alone or their sequential use; however, results from this study are not due until 2015. In the meantime, two agents are currently available for clinical use that, from this analysis, would appear to work better when used in sequence rather than as individual monotherapies. All patients in this analysis received the second of their sequential treatments after disease progression had been documented. It is possible that switching prior to disease progression in BRAF mutation-positive patients, i.e. when the patient has achieved disease control, would result in more durable outcomes. The optimal timing of sequential therapy, however, requires further clinical investigation.

## Conclusions

The results of this preliminary analysis suggest that it may be possible to determine the optimal sequence of treatments in patients with BRAF mutation-positive metastatic melanoma based on presence of specific risk factors; however, further investigation in a larger number of patients is required to validate this hypothesis.

The optimal sequencing paradigm for patients with metastatic melanoma has not yet been fully determined. However, the availability of two new agents that provide an overall survival benefit in phase III clinical trials has brought hope to a therapy area that previously relied on enrolment into a clinical trial as the best option. Optimisation of treatment strategies in the future will provide additional clinical benefit for patients with metastatic melanoma.

## Competing interests

PAA has served as a consultant and advisor for Merck Sharp & Dohme, participated in advisory boards for Bristol-Myers Squibb, Roche-Genentech, GlaxoSmithKline, Amgen, Celgene, Medimmune and Novartis, and received honoraria from Bristol-Myers Squibb, Merck Sharp & Dohme and Roche-Genentech. ES has received honoraria from Bristol-Myers Squibb. DG, AMG, AR and NM declare that they have no competing interests.

## Authors’ contributions

PAA, ES, AMG, AR and NM were study investigators involved in the collection, analysis, and interpretation of data and participated in drafting and revising the manuscript. DG participated in the design and execution of statistical analyses and drafting the manuscript. All authors read and approved the final manuscript.
